# Supplementary material for: Heat adaptation of phage T7 under an extended genetic code
Source: Virus Evol. 2021 Dec 1;7(2):veab100. doi: 10.1093/ve/veab100 (PMC8923235; doi:10.1093/ve/veab100)
Supplement: veab100_Supp [file veab100_supp.zip › Sequencing_Analysis.zip!/Sequencing_Analysis/A3/evidence/SNP_4.html]

BRESEQ :: Evidence


| Predicted mutation | | | | | | |
| --- | --- | --- | --- | --- | --- | --- |
| evidence | seq id | position | mutation | annotation | gene | description |
| RA | NC\_001604.1 | 20,544 | G→A | intergenic (–/–) | – / – | –/– |

| Read alignment evidence... | | | | | | | | | | | |
| --- | --- | --- | --- | --- | --- | --- | --- | --- | --- | --- | --- |
|  | seq id | position | | ref | new | freq | score (cons/poly) | reads | annotation | genes | product |
| \* | NC\_001604.1 | 20,544 | 0 | G | A | 99.7% | 4413.9 / NA | 1929 | intergenic (–/–) | *–/–* | –/– |
| Reads supporting (aligned to +/- strand):  **ref** base G (0/1);  **major** base A (925/994);  **minor** base C (4/2);  **total** (930/999) | | | | | | | | | | | |
| Fisher's exact test for biased strand distribution *p*-value = 4.38e-01 | | | | | | | | | | | |
| Kolmogorov-Smirnov test that lower quality scores support variant *p*-value = 1.00e+00 | | | | | | | | | | | |

Only 100 of 1929 total aligned reads displayed.  

`GAGAAACGAACAGGACTTGCGGAGGATGGCGCAAAGTCTGTCTATGAGCGTTTAAAGAACGACCGTGCTCCCTATGAGACACGCGCTCAGAATTGCGCTCAATATACCATCCCATCATTGTTCCCTAAGGACTCCGATAACGCCTCTACAGATTATCAAACTCCGTGGCAAGCCGTGGGCGCTCGTGGTCTGAACAATCTAGCCTCTAAGCTCATGCTGGCTCTATTCCCTATGCAGACTTGGATGCGACTTACTATATCTGAATATGAAGCAAAGCAGTTACTGAGCGACCCCGATGGACTCGCTAAGGTCGATGAGGGCCTCTCGATGGTAGAGCGTATCATCATGAACTACATTGAGTCTAACAGTTACCGCGTGACTCTCTTTGAGGCTCTCAAACAGTTAGTCGTAGCTGGTAACGTCCTGCTGTACCTACCGGAACCGGAAGGGTCAAACTATAATCCCATGAAGCTGTACCGATTGTCTTCTTATGTGGTCCAACGAGACGCATTCGGCAACGTTCTGCAAATGGTGACTCGTGACCAGATAGCTTTTGGTGCTCTCCCTGAGGACATCCGTAAGGCTGTAGAAGGTC  >  NC_001604.1/20246‑20840`  

                                                                                                                                                                                                                                                                                                          `|`                                                                                                                                                                                                                                                                                                          
    `agatgtgtataagagacagggaTGGCGCAAAGTCTGTCTATGAGCGTTTAAAGAACGACCGTGCTCCCTATGAGACACGCGCTCAGAATTGCGCTCAATATACCATCCCATCATTGTTCCCTAAGGACTCCGATAACGCCTCTACAGATTATCAAACTCCGTGGCAAGCCGTGGGCGCTCGTGGTCTGAATAATCTAGCCTCTAAGCTCATGCTGGCTCTATTCCCTATGCAGACTTGGATGCGACTTACTATATCTGAATATGAAGCAAAGCAGTTACTGAGCGACCCCGATGAACTCGc                                                                                                                                                                                                                                                                                                    <  1:118109/282‑1 (MQ=255)`  
      `cGAACAGGACTTGCGGAGGATGGCGCAAAGTCTGTCTATGAGCGTTTAAAGAACGACCGTGCTCCCTATGAGACACGCGCTCAGAATTGCGCTCAATATACCATCCCATCATTGTTCCCTAAGGACTCCGATAACGCCTCTACAGATTATCAAACTCCGTGGCAAGCCGTGGGCGCTCGTGGTCTGAACAATCTAGCCTCTAAGCTCATGCTGGCTCTATTCCCTATGCAGACTTGGATGCGACTTACTATATCTGAATATGAAGCAAAGCAGTTACTGAGCGACCCCGATGAACTCGCTa                                                                                                                                                                                                                                                                                                  >  1:186784/1‑301 (MQ=255)`  
          `gtataagagacagggaTGGAGCAAAGTCTGTCTATGAGCGTTTAAAGAACGACCGTGCTCCCTATGAGACACGCGCTCAGAATTGCGCTCAATATACCATCCCATAATTGTTCCCTAAGGACTCCGATAACGCCTATACAGATTATCAAACTCCGTGGCAAGCCGTGGGCGCTCGTGGTCTGAACAATCTAGCCTCTAAGCTCATGCTGGCTCTATTCCCTATGCAGACTTGGATGCGACTTACTATATCTGAATATGAAGCAAAGCAGTTACTGAGCGACCCCGATGAACTCGCTAAGGt                                                                                                                                                                                                                                                                                              <  1:160594/288‑1 (MQ=255)`  
               `gtataagagacaggCGCAAAGTCTGTCTATGAGCGTTTAAAGAACGACCGTGCTCCCTATGAGACACGCGCTCAGAATTGCGCTCAATATACCATCCCATCATTGTTCCCTAAGGACTCCGATAACGCCTCTACAGATTATCAAACTCCGTGGCAAGCCGTGGGCGCTCGTGGTCTGAACAATCTAGCCTCTAAGCTCATGCTGGCTCTATTCCCTATGCAGACTTGGATGCGACTTACTATATCTGAATATGAAGCAAAGCAGTTACTGAGCGACCCCGATGAACTCGCTAAGGTCGATg                                                                                                                                                                                                                                                                                         <  1:184349/289‑1 (MQ=255)`  
               `cTTGCGGAGGATGGCGCAAAGTCTGTCTATGAGCGTTTAAAGAACGACCGTGCTCCCGATGAGACACGCGCTCAGAATTGCGCTCAATATACCATCCAATCATTGTTCCCTAAGGACTCCGATAACGCCTCTACAGATTATCAAACTCCGTGGCAAGCCGTGGGCGCTCGTGGTCTGAACAATCTAGCCTCTAAGCTCATGCTGGCTCTATTCCCTATGCAGACTTGGATGCGACTTACTATATCTGAATATGAAGCAAAGCAGTTACTGAGCGACCCCGATGAACTCGCTAAGGTCGATg                                                                                                                                                                                                                                                                                         <  1:195359/301‑1 (MQ=255)`  
                 `tGCGGAGGATGGCGCAAAGTCTGTCTATGAGCGTTTAAAGAACGACCGTGCTCCCTATGAGACACGCGCTCAGAATTGCGCTCAATATACCATCCCATCATTGTTCCCTAAGGACTCCGATAACGCCTCTACAGATTATCAAACTCCGTGGCAAGCCGTGGGCGCTCGTGGTCTGAACAATCTAGCCTCTAAGCTCATGCTGGCTCTATTCCCTATGCAGACTTGGATGCGACTTACTATATCTGAATATGAAGCAAAGCAGTTACTGAGCGACCCCGATGAACTCGCTAAGGTCGATGAg                                                                                                                                                                                                                                                                                       <  1:244361/301‑1 (MQ=255)`  
                  `gCGGAGGATGGCGCAAAGTCTGTCTATGAGCGTTTAAAGAACGACCGTGCTCCCTATGAGACACGCGCTCAGAATTGCGCTCAATATACCATCCCTTCATTGTTCCCTAAGGACTCCGATAACGCCTCTACAGATTATCAAACTCCGTGGCAAGCCGTGGGCGCTCGTGGTCTGAACAATCTAGCCTCTAAGCTCATGCTGGCTCTATTCCCTATGCAGACTTGGATTCGACTTACTATAGCTGAATATGAAGCAAAGCAGTTACTGAGCGACCCCGATGAACTCGCTAATGTCGATGAgg                                                                                                                                                                                                                                                                                      >  1:340712/1‑301 (MQ=255)`  
                     `gaggaTGGCGCAAAGTCTGTCTATGAGCGTTTAAAGAACGACCGTGCTCCCTATGAGACACGCGCTCAGAATTGCGCTCAATATACCATCCCATCATTGTTCCCTAAGGACTCCGATAACGCCTCTACAGATTATCAAACTCCGTGGCAAGCCGTGGGCGCTCGTGGTCTGAACAATCTAGCCTCTAAGCTCATGCTGGCTCTATTCCCTATGCAGACTTGGATGCGACTTACTATATCTGAATATGAAGCAAAGCAGTTACTGAGCGACCCCGATGAACTCGCTAAGGTCGATGAGGGcc                                                                                                                                                                                                                                                                                   <  1:275365/301‑1 (MQ=255)`  
                          `tGGCGCAAAGTCTGTCTATGAGCGTTTAAAGAACGACCGTGCTCCCTATGAGACACGCGCTCAGAATTGCGCTCAATATACCATCCCATCATTGTTCCCTAAGGACTCCGATAACGCCTCTACAGATTATCAAACTCCGTTGCAAGCCGTGGGCGCTCGTGGTCTGAACAATCTAGCCTCTAAGCTCATGCTGGCTCTATTCCCTATGCAGACTTGGATGCGACTTACTATATCTGAATATGAAGCAAAGCAGTTACTGAGCGACCCCGATGAACTCGCTAAGGTCGATGAGGGCCTCTCg                                                                                                                                                                                                                                                                              <  1:82299/301‑1 (MQ=255)`  
                            `gacacaaGTCTGTTTACGAGCGTTTAAAGACCGACCGTGCTACCTATGAGACACGCGCTCATACTTGCGCTCAATATACCATCATATCATTGTTCCCTAAGGACTCCGATACCGCCTCTACAGATTATCAAACTCCGTGGCAAGCCGTGGGCGCTCGTGGTCTGAACAATCTAGCCTCTAAGCTTATGCTGGCTCTATTCCCTATGCAGACTTGGATGCCACTTCCTATATCTGAATATGAAGCAAAGCAGTTACTGAGCGACCCCGATGAACTCGCTAAGGTCGATGAGGGCCTCTCGAt                                                                                                                                                                                                                                                                            <  1:335143/296‑1 (MQ=255)`  
                                 `aaGTCTGTCTATGAGCGTTTAAAGACCGACCGTGCTCCCTATGAGACACGCGCTCAGAATTGCGCTCAATATACCATCCCATCATTGTTCCCTAAGGACTCCGATAACGCCTCTACAGATTATCAAACTCAGTGGCAAGCCGTGGGCGCTCGTGGTCTGAACAATCTAGCCTCTAAGCTCATGCTGGCTCTATTCCCTATGCAGACTTGGATGCGACTTACTATATCTGAATATGAAGCAAAGCAGTTACTGAGCGACCCCGATGAACTCGCTAAGGTCGATGAGGGCCTCTCGATGGTag                                                                                                                                                                                                                                                                       <  1:149571/301‑1 (MQ=255)`  
                                   `gtctgtctATGAGCGTTTAAAGAACGACCGTGCTCCCTATGAGACACGCGCTCAGAATTGCGCTCAATATACCATCCCATCATTGTTCCCTAAGGACTCCGATAACGCCTCTACAGATTATCAAACTCAGTGGAAAGCCGTGGGCGCTCGTGGTCTGAACAATCTAGCCTCTAAGCTCATGCTGGCTCTATTCCCTATGCAGACTTGGCTGCGACTTACTATATCTGAATATGAAGCAAAGCAGTTACTGAGCGACCCCGATGAACTAGCTAAGGTCGATGAGGGCCTCTCGATGGTagag                                                                                                                                                                                                                                                                     >  1:222864/1‑301 (MQ=255)`  
                                   `gtctgtctATGAGCGTTTAAAGAACGACCGTGCTCCCTATGAGACACGCGCTCAGAATTGCGCTCAATATACCATCACATCATTGTTCCCTAAGGACTCCGATAACGCCTCTACAGATTATCAAACTCCGTGGCAAGCCGTGGGCGCTCGTGGTCTGAACAATCTAGCCTCTCAGCTCATGCTGGCTCTATTCCCTATGCAGACTTGGATGCGACTTACTATATCTGAATATGAAGCAAAGCAGTTACTGAGCGACCCCGATTAACTCGCTCAGGTCGATGAGGGCCTCTCGATGGTcgag                                                                                                                                                                                                                                                                     >  1:236424/1‑301 (MQ=255)`  
                                   `acaggtctAGGAGCGTTAAAAGAACGACCGTGATCCCTATGAGACACGCGCTCAGAATTGCGCTCAATATACCATCCCATCAGTGTTCCCTAAGGACTCCGATAACGCCTCTACAGATTATCAAACTCCGTGGCAAGCCGTGGGCGCTCGTGGTCTGAACAATCTAGCCTCTAAGCTCATGCTGGCTCTATTCCCTATGCAGACGTGGATGCGACTTACTATATCTGAATATGAAGCAAAGCAGTTACTGAGCGACCCCGATGAACTCGCTAAGGTCGATGAGGGCCTCTCGATGGTagag                                                                                                                                                                                                                                                                     <  1:228558/297‑1 (MQ=255)`  
                                    `tctgtctATGAGCGTTTAAAGAACGACCGTGCTCCCTATGAGACACGCGCTCAGAATTGCGCTCAATATACCATCCCATCATTGTTCCCTAAGGACTCCGATAACGCCTCTACAGATTATCAAACTCAGTGGCAAGCCGTGGGCGCTCGTGGTCTGAACAATCTAGCCTCTAAGCTCATGCTGGCTCTATTCCCTATGCAGACTTGGATGCGACTTACTATATCTGAATATGAAGCAAAGCAGTTACTGAGCGACCCCGATGAACTCGCTAAGGTCGATGAGGGCCTCTCGATGGTAGAGc                                                                                                                                                                                                                                                                    <  1:191379/301‑1 (MQ=255)`  
                                     `ctgtctATGAGCGTTTAAAGAACGACTGTGCTCCCTATGAGACACGCGCTCAGAATTGCGCTCAATATACCATCCCATCATTGTTCCCTAAGGACTCCGATAACGCCTCTACAGATTATCAAACTCCGTGGCAAGCCGTGGGCGCTCGTGGTCTGAACAATCTAGCCTCTAAGCTCATGCTGGCTCTATTCCCTATGCAGACTTGGATGCGACTTACTATATCTGAATATGAAGCAAAGCAGTTACTGAGCGACCCCGATGAACTCGCTAAGGTCGATGAGGGCCTCTCGATGGTAGAGCg                                                                                                                                                                                                                                                                   <  1:302929/301‑1 (MQ=255)`  
                                       `gtctATGAGCGTTTAAAGAACGACCGTGCTCCCTATGAGACACGCGCTCAGAATTGCGCTCAATATACCATCCCATCATTGTTCCCTAATGACTCCGATAACGCCTCTACAGATTATCAAACTCCGTGGCAAGCCGTGGGCGCTCGTGGTCTGAACAATCTAGCCTCTAAGCTCATGCTGGCTCTATTCCCTATGCAGACTTGGATGCGACTTACTATATCTGAATATGAAGCAAAGCAGTTACTGAGCGACCCCGATGAACTCGCTAAGGTCGATGAGGGCCTCTCGATGGTagctgtct                                                                                                                                                                                                                                                                 >  1:319796/1‑295 (MQ=255)`  
                                       `gtctATGAGCGTTTAAAGAACGACCGTGCTCCCTATGAGACACGCGCTCAGAATTGCGCTCAATATACCATCCCATCATTGTTCCCTAAGGACTCCGATAACACCTCTACAGATTATCAAACTCCGTGGCAAGCCGTAGGCGCTCGTGGTCTGAACAATCTAGCCTCTAAGCTCATGCTGGCTCTATTACCTATGCAGACTTGGATGCGACTTACTATATCTGTATATGAAGCAAAGCAGTTACTGAGCGACCACGATGAACTCGCTAAGGTCGATGAGGGCCTCTCGATGGTAGAGCGTa                                                                                                                                                                                                                                                                 >  1:145294/1‑301 (MQ=255)`  
                                       `gtctATGAGCGTTTAAAGAACGACCGTGCTCCCTATGAGACACGCGCTCAGAATTGCGCTCAATATACCATCCCATCATTGTTCCATAAGGACTCCGATAACGCCTATACAGATTATCAAACTCCGTGGCAAGCCGTGGGCGCTCGTGGTCTGAACAATCTAGCCTCTAAGCTCATGCTGGCTCTATTCCCTATGCAGACTTGGATGCGACTTACTATATCTGAATATGAAGCAAAGCAGTTACTGAGCGACCCCGATGAACTCGCTAAGGTCGATGAGGGCCTCTCGATGGTAGAGCGTa                                                                                                                                                                                                                                                                 >  1:104001/1‑301 (MQ=255)`  
                                       `gtctATGAGCGTTTAAAGAACGACCGTGCTCCCTATGAGACACGCGCTCAGAATTGCGCTCAATATACCATCCAATCATTGTTCCCTAAGGACTCCGATAACGCCTCTACAGATTATCAAACTCCGTGGCAAGCCGTGGGCGCTCTTGGTCTGAACAATCTAGCCTCTAAGCTCATGCTGGCTCTATTCCCTATGCCGACTTGGTTGCGACTTACTATATCTGAATATGAAGCAAAGCAGTTACTGAGCGACCCCGATGAACTCGCTAAGGTCGATGAGGGCCTCTCGATGGTAGAGctgt                                                                                                                                                                                                                                                                 >  1:239831/1‑298 (MQ=255)`  
                                           `aTGAGCGTTTAAAGAACGACCGTGCTCCCTATGAGAAACGCGCTCAGAATTGCGCTCAATATACCATCCCATCATTGTTCCCTAAGGACTCCGATAACGCCTCTACAGATTATCAAACTCCGTGGCAAGCCGTGGGCGCTCGTGGTCTGAACAATCTAGCCTCTAAGCTCATGCTGGCTCTATTCCCTATGCAGACTTGGATGCGACTTACTATATCTGAATATGAAGCAAAGCAGTTACTGAGCGACCTCGATGAACTCGCTAAGGTCGATGAGGGCCTCTCGATGGTAGAGCGTatcat                                                                                                                                                                                                                                                             <  1:286931/301‑1 (MQ=255)`  
                                                     `aaaGTACGACCGTGCTCCCTATGCGACACGCGCTCAGAATTGCGCTCAATATACCATCCCATCATTGTTCCCTAAGGACTCCGATAACGCCTCTACAGATTATCAAACTCCGTGGCAAGCCGTGGGCGCTCGTGGTCTGAACAATCTAGCCTCTAAGCTCATGCTGGCTCTATTCCCTATGCAGACTTGGATGCGACTTACTATATCTGAATATGAAGCAAAGCAGTTACTGAGCGACCCCGATGAACTCGCTAAGGTCGATGAGGGCCTCTCGATGGTAGAGCGTATCATCATGAACTAc                                                                                                                                                                                                                                                   <  1:181093/301‑1 (MQ=255)`  
                                                       `agatgtgtagaagagacagtGAGACACGCGCTCAGAATTGCGCTCAATATACCATCCCATCATTGTTCCCTAAGGACTCCGATAACGCCTCTACAGATTATCAAACTCCGTGGCAAGCCGTGGGCGCTCGTGGTCTGAACAATCTAGCCTCTAAGCTCATGCTGGCTCTATTCCATATGCAGACTTGGATGCGACTTACTATATCTGAATATGAAGCAAAGCAGTTACTGAGCGACCCCGATGAACTCGCTAAGGTCGATGAGGGCCTCTCGATGGTAGAGCGTATCATCATGAACTACAt                                                                                                                                                                                                                                                 <  1:210190/282‑1 (MQ=255)`  
                                                        `gAACGACCGTGCTCCCTATGAGACACGCGCTCAGAATTGCGCTCAATATACCATCCCATCATTGTTCCCTAAGGACTCCGATAACGCCTCTACAGATTATCAAACTCCGTGGCAAGCCGTGGGCGCTCGTGGTCTGAACAATCTAGCCTCTAAGCTCATGCTGGCTCTATTCCCTATGCAGACTTGGATGCGACTTACTATATCTGAATATGAAGCAAAGCAGTTACTGAGCGACCCCGATGAACTCGCTAAGGTCGCTGAGGGCCTCTCGATGGTAGAGCGTATCATCATGAACTACAtt                                                                                                                                                                                                                                                >  1:172508/1‑301 (MQ=255)`  
                                                                  `gCTCCCTATGAGACACGCGCTCAGAATTGCGCTCAATATACCATCCCATCATTGTTCCCTAAGGACTCCGATAACGCCTCTACAGATTATCAAACTCCGTGGCAAGCCGTGGGCGCTCGTGGTCTGAACAATCTAGCCTCTAAGCTCATGCTGGCTCTATTCCCTATGCAGACTTGGATGCGACTTACTATATCTGAATATGAAGCAAAGCAGTTACTGAGCGACCCCGATGAACTCGCTAAGGTCGATGAGGGCCTCTCGATGGTAGAGCGTATCATCATGAACTACATTGAGTCtgtct                                                                                                                                                                                                                                      >  1:111493/1‑297 (MQ=255)`  
                                                                     `cccTATGAGACACGCGCTCAGAATTGCGCTCAATATACCATCCCATCATTGTTCCCTAAGGACTCCGATAACGCCTCTACAGATTATCAAACTCCGTGGCAAGCCGTGGGCGCTCGTGGTCTGAACAATCTAGCCTCTAAGCTCATGCTGGCTCTATTCCCTATGCAGACTTGGATGCGACTTACTATATCTGAATATGACGCAAAGCAGTTACTGAGCGACCCCGATGAACTCGCTAAGGTCGATGAGGGCCTCTCGAGGGTCGAGCGTATCATCATGAACTACATTGAGTAGAACAggt                                                                                                                                                                                                                                   >  1:231691/1‑299 (MQ=255)`  
                                                                     `cccTATGAGACACGCGCTCAGAATTGCGCTCAATATACCATCCCATCATTGTTCCCTAAGGACTCCGATAACGCCTCTACAGATTATCAAACTCCGTGGCAAGCCGTAGGCGCTCGTGGTCTTAACAATCTAGCCTCTAAGCTCATGCTGGCTCTATTCCCTATGCAGACTTTGATGCGACTTACTATATCTGAATATGAAGAAAAGCAGTTACTGAGCGTCCCCGATGAACTCGCTAAGGTCGATTAGGGCCTCTCGATGGTAGAGCGCATCATCATGAACTACATTGAGTCTAAAAGtt                                                                                                                                                                                                                                   >  1:141166/1‑301 (MQ=255)`  
                                                                         `aTGAGACACGCGCTCAGAATTGCGCTCAATATACCATCCCATCATTGTTCCCTAAGGACTCCGATAACGCCTCTACAGATTATCAAACTCCGTGGCAAGCCGTGGGCGCTCGTGGTCTGAACAATCTAGCCTCTAAGCTCATGCTGGCTCTATTCCCTATGCAGACTTGGATGCGACTTACTATATCTGAATATGAAGCAAAGCAGTTACTGAGCGACCCCGATGAACTCGCTAAGGTCGATGAGGGCCTCTCGATGGTAGAGCGTATCATCATGAACTACATTGAGTCTAACAGTTACcg                                                                                                                                                                                                                               <  1:233598/301‑1 (MQ=255)`  
                                                                                     `cTCCGAATTGCGCTCAATATACCATCCCATCATTGTTCCCTAAGGACTCCGATAACGCCTCTACAGATTATCAAACTCCGTGGCAAGCCGTGGGCGCTCGTGGTCTGAACAATCTAGCCTCTAAGCTCATGCTGGCCCTATTCCCTATGCAGACTTGGTTGCGACTTACTATATCTGTATATGAAGCAAAGCAGTTACTGAGCGACTCCGATGAACTCGCTAAGGTCGATGAGGGCCTCTCGATGGTAGAGCGTACCATCATGAACTACATCGAGTCTAACAGTTACCGCGTGACTCTCtt                                                                                                                                                                                                                   >  1:284157/1‑301 (MQ=255)`  
                                                                                       `cAGAATTGCGCTCAATATACCCTCCCATCATTGTTCCCTAAGGACTCCGATAACGCCTCTACAGATTATCAAACTCCGTGGCAAGCCGTGGGCGCTCGTGGTCTGAACAATCTAGCCTCTAAGCTCATGCTGGCTCTATTCCCTATGCAGACTTGGATGCGACTTACTGTATCTGAATATGAAGCAAAGCAGTTACTGAGCGACCCCGATGAACTCGCTAAGGTCGATGAGGGCCTCTCGATGGTAGAGCGTATCATCATGAACTACATTGAGTCTAACAGTTACCGCGTGACTCTCTTTg                                                                                                                                                                                                                 <  1:306072/301‑1 (MQ=255)`  
                                                                                              `gcgcTCAATATACCATCCCATCATTGTTCACTAAGGACTCCGATAACGCCTCTACAGATTATCAAACTCCGTGGCAAGCCGTGGGCGCTCGTGGTCTGAACAATCTAGCATCTAAGCTCATGCTGGCTCTATTCCCTATGCAGACTTGGATGCGACTTACTATATCGGAAGATGAAGCAAAGCAGTTACTGAGCGACCCCGATGAAATCGCTAAGGTCGATGAGGGCCTCTCGATGGTAGAGCGTATCATCATGAACTACATTTAGTCTAACAGGTACCGCGTGACTCTCTTTGAGGctct                                                                                                                                                                                                          >  1:35642/1‑301 (MQ=255)`  
                                                                                                 `cTCAATATACCATCCCATCATTGTTCCCTAAGGACTCCGATAACGCCTCTACAGATTATCAAACTCCGTGGCAAGCCGTGGGCGCTCGTGGTCTGAACAATCTAGCCTCTAAGCTCATGCTGGCTCTATTCCCTATGCAGACTTGGATGCGACTTACTATATCTGAATATGAAGCAAAGCAGTTACTGAGCGACCCCGATGAACTCGCTAAGGTCGATGAGGGCCTCTCGATGGTAGAGCGTATCATCATGAACTACATTGAGTCTAACAGTTACCGCGTGACTCCCTTTGAGGCTCTaaa                                                                                                                                                                                                       >  1:281588/1‑301 (MQ=255)`  
                                                                                                 `agcAATATACCATCCCATCATTGTTCCCTAAGGACTCCGATAACGCCTCTACAGATTATCAAACTCCGTGGCAAGCCGTGGGCGATCGTGGTCTGAACAATCTAGCCTCTAAGCTCATGCTGGCTCTATTCCCTATGCAGACTTGGATGCGACTTACTATATCTGAATATGAAGCAAAGCAGTTACTGAGCGACCCCGATGAACTAGCTAAGGTCGATGAGGGCCTCTCGATGGTAGAGCGTATCATCATGAACTACATTGAGTCTAACAGTTACCGCGTGACTCTCTTTGAGGCTCTCaa                                                                                                                                                                                                       <  1:295896/299‑1 (MQ=255)`  
                                                                                                   `cAATATACCATCCCATCATTGTTCCCTAAGGACTCCGATAACGCCTCTACAGATTATCAAACTCCGTGGCAAGCCGTGGGCGCTCGTGGTCTGAACAATCTAGCCTCTAAGCTCATGCTGGCTCTATTCCCTATGCAGACTTGGATGCGACTTACTATATCTGAATATGAAGCAAAGCAGTTACTGAGCGACCCCGATGAACTCGCTAAGGTCGATGAGGGCCTCTCGATGGTAGATCGTATCATCATGAACTACATTGAGTCTAACAGTTACCGCGTGACTCTCTTTGAGGCTCTCAAAc                                                                                                                                                                                                     >  1:331884/1‑301 (MQ=255)`  
                                                                                                   `cAATATACCATCCCATCATTGTTCCCTAAGGACTCCGATAACGCCTCTACAGATTATCAAACTCCGTGGCAAGCCGTGGGCGCTCGTGGTCTGAACAATCTAGCCTCTAAGCTCATGCTGGCTCTATTCCCTATGCAGACTTGGATGCGACTTACTATATCTGAATATGAAGCAAAGCAGTTACTGAGCGACCCCGATGAACTCGCTAAGGTCGATGAGGGCCTCTCGATGGTAGAGCGTATCATCATGAACTACATTGAGTCTAACAGTTCCCGCGTGACTCTCTTTGAGGCTCTCCAAc                                                                                                                                                                                                     >  1:164632/1‑301 (MQ=255)`  
                                                                                                   `cAATATACCATCCCATCATTGTTCCCTAAGGACTCCGATAACGCCTCTACAGATTATCAAACTCCGTGGCAAGCCGTGGGCGCTCGTGGTCTGAACAATCTAGCCTCTAAGCTCATGCTGGCTCTATTCCCTATGCAGACTTGGATGCGACTTACTATATCTGAATATGAAGCAAAGCAGTTACTGAGCGACCCCGATGAACTCGCTAAGGTCGATGAGGGCCTCTCGATGGTAGAGCGTATCATCATGAACTACATTGAGTCTAACAGTTACCGCGTGACCCTCTTTGAGGCTCTCAAAc                                                                                                                                                                                                     >  1:337669/1‑301 (MQ=255)`  
                                                                                                   `cAATATACCATCCCATCATTGTTCCCTAAGGACTCCGATAACGCCTCTACAGATTATCAAACTCCGTGGCAAGCCGTGGGCGCTCGTGGTCTGAACAATCTAGCCTCTAAGCTCATGCTGGCTCTATTCCCTATGCAGACTTGGATGCGACTTACTATATCTGAATATGAAGCAAAGCAGTTACTGAGCGACCCCGATGAACTCGCTAAGGTCGATGAGGGCCTCTCGATGGTAGAGCGTATCATCATGAACTACATAGCGTCTAACAGATACCGCGTGACTCTCTTTGAGGCTCTCaaaa                                                                                                                                                                                                     >  1:100403/1‑300 (MQ=255)`  
                                                                                                     `gatgtgtataagagacaggTTCCCTAAGGACTCCGATAACGCCTCTACAGATTATCAAACTCCGTGGCAAGCCGTGGGCGCTCGTGGTCTGAACAATCTAGCCTCTAAGCTCATGCTGGCTCTATTCCCTATGCAGACTTGGATGCGACTTACTATATCTGAATATGAAGCAAAGCAGTTACTGAGCGACCCCGATGAACTCGCTAAGGTCGATGAGGGCCTCTCGATGGTAGAGCGTATCATCATGAACTACATTGAGTCTAACAGTTACCGCGTGACTCTCTTTGAGGCTCTCAAACAg                                                                                                                                                                                                   <  1:3285/283‑1 (MQ=255)`  
                                                                                                     `gagatgtgtataagagccagtCCCTAAGGACTCCGATAACGCCTCTACAGATTATCAAACTCCGTGGCAAGCCGTGGGCGCTCGTGGTCTGAACAATCTAGCCTCTAAGCTCATGCTGGCTCTATTCCCTATGCAGACTTGGATGCGACTTACTATATCTGAATATGAAGCAAAGCAGTTACTGAGCGACCCCGATGAACTCGCTAAGGTCGATGAGGGCCTCTCGATGGTAGAGCGTATCATCATGAACTACATTGAGTCTAACAGTTACCGCGTGACTCTCTTTGAGGCTCTCAAACAg                                                                                                                                                                                                   <  1:261936/281‑1 (MQ=255)`  
                                                                                                       `ataCCATCCCATCATTGTTCCCTAAGGACTCCGATAACGCCTCTACAGATTATCAAACTCCGTGGCAAGCCGTGGGCGCTCGTGGTCTGAACAATCTAGCCTCTAAGCTCATGCTGGATCTATTCCCTATGCAGACTTGGATGCGACTTACTATATCTGAATATGAAGCAAAGCAGTTACTGAGCGACCCCGATGAACTCGCTAAGGTCGATGAGGGCCTCTCGATGGTAGAGAGTATCATCATGAACTACATTGAGTCTAACAGTTACCGCGTGACTCTCTTTGAGGCTCTCAAAcctgt                                                                                                                                                                                                 >  1:293013/1‑297 (MQ=255)`  
                                                                                                                `ataagagacagccTAAGGACTCCGATAACGCCTCTACAGATTATCAAACTCCGTGGCAAGCCGTGGGCGCTCGTTGTCTGAACAATCTAGCCTCTAAGCTAATGCTGGCTCTATTCCCTATGCAGACTTGGATGCGACTTACTATATCTGAATATGAAGCAAAGCAGTTACTGAGCGACCCCGATGAACTCGCTAAGGTCGATGAGGGCCTCTCGATGGTAGAGCGTATCATCATGAACTACATTGAGTCTAACAGTTACCGCGTGACTCTCTTTGAGGCTCTCAAACAGTTAGTCGTAGc                                                                                                                                                                                        <  1:127442/290‑1 (MQ=255)`  
                                                                                                                  `gagacagtCCCTAAGGACTCCGATAACGCCTCTACAGATTATCAAACTCCGTGGCAAGCCGTGGGCGCTCGTGGTCTGAACAATCTAGCCTCTAAGCTCATGCTGGCTCTATTCCCTATGCAGACTTGGATGCGACTTACTATATCTGAATATGAAGCAAAGCAGTTACTGAGCGACCCCGATGAACTCGCTAAGGTCGATGAGGGCCTCTCGATGGTAGAGCGTATCATCATGAACTACATTGAGTCTAACAGTTACCGCGTGACTCTCTTTGAGGCTCTCAAACAGTTAGTCGTAGCTg                                                                                                                                                                                      <  1:20191/294‑1 (MQ=255)`  
                                                                                                                          `cccGAAGGACTCCGATAACGCCTCTACATATTATCAAACTCCGTGGCAAGCCGTGGGCGCTCGTGGTCTGAACAATCTAGCCTCTAAGCTCATGCTGGCTCTATTCCCTATGCAGACTTGGATGCGACTTACTATATCTGAATATGAAGCAAAGCAGTTACTGAGCTACCCCGATGAACTCGCTAAGGTCGATGAGGGCCTCTCGATGGTAGAGCGTATCATCATGAACTACATTGAGTCTAACAGTTACCGCGTGACTCTCTTTGAGGCTCTCAAACAGTTAGTCGTAGCTGGTAACGTc                                                                                                                                                                              <  1:29954/301‑1 (MQ=255)`  
                                                                                                                                 `gACTCCTATAACGCCTCTACAGATTATCAAACTCCGTGGCAAGCCGTGGGCGCTCGTGGTCTGAACAATCTAGCCTCTAAGCTCATGCTGGCTCTATTCCCTATGCAGACTTGGATGCGACTTACTATATCTGAATATGAAGCAAAGCAGTTACTGAGCGACCCCGATGAACTCGCTAAGTTCGATGAGGGCCTCTCGATGGTAGAGCGTATCATCATGAACTACATTGAGTCTAACAGTTACCGCGTGACTCTCTTTGAGGCTCTCAAAAAGTTAGTCGTAGCTGGTAACGTCCTGCTGt                                                                                                                                                                       >  1:95714/1‑301 (MQ=255)`  
                                                                                                                                   `tgtatgagaggcaactACATATTAACAAACTCCGTGGCAAGCCGTGGGCGCTCGTGGTCGGAACAATCTAGCATCTAAGCTCATGCTGGCTCTATTCCCTATGCAGACATGGATGCGACTTACTATATCTGAATATGAAGCAAAGCAGTTACTGAGCGACCCCGATGAACTCACTAAGGTCGATGAGTGCCTCTCGATGGTAGGGCGTATCATCATGAACTACATTGAGTCTAACAGTTACCGCGTGGCTCTCTTTGAGGCTCTCAAACAGTTAGTCGTAGCTGGTAACGTCCTGCTGtac                                                                                                                                                                     <  1:134722/287‑1 (MQ=255)`  
                                                                                                                                   `cTCCGATAACGCCTCTACAGATTATCAAACTCCGTGGCAAGCCGTGGGCGCTCGTGGTCTGAACAATCTAGCCTCTAAGCTCATGCTGGCTCTATTCCCTATGCAGACTTGGATGCGACTTACTATATCTGAATATGGAGCAAAGCAGTTACTGAGCGACCCCGATGAACTCGCTAAGGTCGATGAGGGCCTCTCGATGGTAGAGCGTATCATCATGAACTACATTGAGTCTAACAGTTACCGCGTGACTCTCTTTGAGGCTCTCAAACAGTTAGTCGTAGCTGGtctgtcacttatacaa                                                                                                                                                                     >  1:136854/1‑286 (MQ=255)`  
                                                                                                                                     `ccGATAACGCCTCTACAGATTATCAAACTCCGTGGCAAGCCGTGGGCGCTCGTGGTCTGAACAATCTAGCCTCTAAGCTCATGCTGGCTCTATTCCCTATGCAGACTTGGATGCGACTTACTATATCTGAATATGAAGCAAAGCAGTTACTGAGCGACCCCGATTAACTCGCTAAGGTCGATGAGGGCCTCTCGATGGTAGAGCGTATCATCATGAACTACATTGAGTCTAACAGTTACCGCGTGACTCTCTTTGAGGCTCTCAAACAGTTAGTCGTAGCTGGTAACGTCCTGCTGtacct                                                                                                                                                                   <  1:215090/301‑1 (MQ=255)`  
                                                                                                                                       `gATAACGCCTCTACAGATTATCAAACTCCGTGGCAAGCCGTGGGCGCTCGTGGTCTGAACAATCTAGCCTCTAGGCTCATGCTGGCTCTATTACCTATGCAGACTTGGATGCGACTTACTATATCTGAATATGAAGCAAAGCAGTTACTGAGCGACCCCGATGAACTCGCTAAGGTCGATGAGGGCCTCTAGATGGTAGAGCGTATCATCATGAACTACATTGAGTCTAACAGTTACCGCGTGACTCTCTTTGAGGCTCTCAAACAGTTAGTCGTAGCTGGTAACGTCCTGCTGtacctac                                                                                                                                                                 <  1:248206/301‑1 (MQ=255)`  
                                                                                                                                       `gATAACGCCTCTACAGATTATCAAACTCCGTGGCAAGCCGAGGGCGCTCGTGGTCTGAACAATCTAGCCTCTAAGCTCATGCTGGCTCTATTCCCTATGCAGACTTGGATGCGACTTACTATATCTGAATATGAAGCAAAGCAGTTACTGAGCGACCCCGATGAACTCGCTAAGGTCGATGAGGGCCTCTCGATGGTAGAGCGTATCATCATGAACTACATTGAGTCTAACAGTTACCGCGTGACTCTCTTTGAGGCTCTCAAACAGTTAGTCGTAGCTGGTAACGTCCTGCTGtacctac                                                                                                                                                                 >  1:278259/1‑301 (MQ=255)`  
                                                                                                                                                        `ttATCAAACTCCGTGGCAAGCCGTGGGCGCTCGTGGTCTGAACAATCTAGCCTCTAAGCTCATGCTGGCTCTATTCCCTATGCAGACTTGGATGCGACTTACTATATCTGCATATGAAGCAAAGCAGTTACTGAGCGACCCCGATGAACTCGCTAAGGTCGATGAGGGCCTCTCGATGGTAGAGCGTATCATCATGAACTACATTGAGTCTAACAGTTACCGCGTGACTCTCTTTGAGGCTCTCAAACAGTTAGTCGTAGCTGGTAACGTCCTGCTGTACCTACCGGAACCGGTATGGTCa                                                                                                                                                >  1:53458/1‑301 (MQ=255)`  
                                                                                                                                                        `ttATCAAACTCCGTGGCAAGCCGTGGGCGCTCGTGGTCTGAACAATCTAGCCTCTAAGCTCATGCTGGCTCTATTCCCTATGCAGACTTGGATGCGACTTACTATATCTGAATTTGAAGCAAAGCAGTTACTGAGCGACCCCGATGAACTAGCTAAGGTCGATGAGGGCCTCTCGATGGTAGCGCGTATCATCATGAACTACATTGAGTCTAACAGTTACTGCGTGACTCTCTTTGAGGCTCTCAAACAGTTAGTCGTAGCTGGTAACGTCCTGCTGTACCTTCCGGAACCCGAAgggctg                                                                                                                                                >  1:205295/1‑298 (MQ=255)`  
                                                                                                                                                        `ttATCAAACTCCGTGGCAAGCCGTGGGCGCTCGTGGTCTGAACAATCTAGCCTCTAAGCTCATGCTGGCTCTATTCCCTATGCAGACTTGGATGCGACTTACTATATCTGAATATGAAGCAAAGCAGTTACTGAGCGACCCCGATGAACTCGCTAAGGTCGATGAGGGCCTCTCGATGGTAGAGCGTATCATCATGAACTACATTGAGTCTACCAGTTACCGCGTGACTCTCTTTGAGGCTCTCAAACAGTTAGTCGTAGCTGGTAACGTCCTGCTGTACCTACCGGAACCGGAAGGGGCa                                                                                                                                                >  1:1254/1‑301 (MQ=255)`  
                                                                                                                                                        `ttATCAAACTCCGTGGCAAGCCGTGGGCGCTCGTGGTCTGAACAATCTAGCCTCTAAGCTCATGCTGGCTCTATTCCCTATGCAGACTTGGATGCGACTTACTATATCTGAATATGAAGCAAAGCAGTTACTGAGCGACCCCGATGAACTCGCTAAGGTCGATGAGGGCCTCTCGATGGTAGAGCGTATCATCATGAACTACATTGAGTCTAACAGTTACCGCGTGACTCTCTTTGAGGCTCTCAAACAGTTAGTCGTAGCTGGTAACGTCCTGCTGTACCTACCGGAACCGGAAGGGTCa                                                                                                                                                >  1:65597/1‑301 (MQ=255)`  
                                                                                                                                                        `ttATCAAACTCCGTGGCAAGCCGTGGGCGCTCGTGGTCTGAACAATCTAGCCTCTAAGCTCATGCTGGCTCTATTCACTATGCAGACTTGGATGCGACTTACTATATCTGAATATGAAGCAAAGCAGTTACTGAGCGACCCCGATGAACTCGCTAAGGTCGATGAGGGCCTCTCGATGGTAGAGCGTATCATCATGAACTACATTGAGTCTAACAGTTACCGCGTGACTCTCTTTGAGGCTCTCAAACAGTTAGTCGTAGCTGGTAACGTCCTGCTGTACCTACCGGAACCGGACGGGTCa                                                                                                                                                >  1:271700/1‑301 (MQ=255)`  
                                                                                                                                                            `aaaaCTCCGTGGCAAGCCGTGGGCGCTCGTGGTCTGAACAATCTAGCCTCTAAGCTCATGCTGGCTCTATTCCCTATGCAGACTTGGATGCGACTTACTATATCTGAATATGAAGCAAAGCAGTTACTGAGCGACCCCGATGAACTCGCTAAGGTCGATGAGGGCCTCTCGATGGTAGAGCGTATCATCATGAACTACATTGAGTCTAACAGTTACCGCGTGACTCTCTTTGAGGCTCTCAAACAGTTAGTCGTAGCTGGTAACGTCCTGCTGTACCTACCGGAACCGGAAGGGTCAAACt                                                                                                                                            <  1:71857/300‑1 (MQ=255)`  
                                                                                                                                                               `aCTCCGTTGCAAGCCGTGGGCGCTCGTGGTCTGAACAATCTAGCCTCTAAGCTCATGCTGGCTCTATTCCCTATGCAGACTTGGATGCGACTTACTATATCTGAATATGAAGCAAAGCAGTTACTGAGCGACCCCGATGAACTCGCTAAGGTCGATGAGGGCCTCTCGATGGTAGAGCGTATCATCATGAACTACATTGAGTCTAACAGTTACCGCGTGACTCTCTTTGAGGCTCTCAAACAGTTAGTCGTAGCTGGTAACGTCCTGCTGTACCTaccggcaccggcactgtctcttatac                                                                                                                                         >  1:92346/1‑286 (MQ=255)`  
                                                                                                                                                                   `cGTGGCAAGCCGTGGGCGCTCGTGGTCTGAACAATCTAGCCTCTAAGCTCATGCTGGCTCTATTCCCTATGCAGACTTGGATGCGACTTACTATATCTGAATATGAAGCAAAGCAGTTACTGAGCGACCCCGATGAACTCGCTAAGGTCGATGAGGGCCTCTCGATGGTAGAGCGTATCATCATGAACTACATTGAGTCTAACAGTTACCGCGTGACTCTCTTTGAGGCTCTCAAACAGTTAGTCGTAGCTGGTAACGTCCTGCTGTACCTACCGGAACCGGAAGGGTCAAACTATAATcc                                                                                                                                     <  1:85340/301‑1 (MQ=255)`  
                                                                                                                                                                   `cGTGGCAAGCCGTGGGCGCTCGTGGTCTGAACAATCTAGCCTCTAAGCTCATGCTGGCTCTATTCCCTATGCAGACTTGGATGCGACTTACTATATCTGAATAGGAAGCAAAGCAGTTACTGAGCGACGCCGATGAACTCGCTAAGGTCGATGAGGGCCTCTCGATGGTAGAGCGTATCATCATGAACTACATTGAGTCTAACAGTTACCGCGTGACTCTCTTTGAGGCTCTCAAACAGTTAGTCGTAGCTGGTAACGTCCTGCTGTACCTACCGGAACCGGAAGGGTCAAACTATAATcc                                                                                                                                     <  1:268987/301‑1 (MQ=255)`  
                                                                                                                                                                      `ggCAAGCCGTGGGCGCTCGTGGTCTGAACAATCTAGCCTCTAAGCTCATGCTGGCTCTATTCCCTATGCAGACTTGGATGCGACTTACTATATCTGAATATGAAGCAAAGCAGTTACTGAGCGACCCCGATGAACTCGCTAAGGTCGATGAGGGCCTCTCGATGGTAGAGCGTATCATCATGAACTACATTGAGTCTAACAGTTACCGCGTGACTCTCTTTGAGGCTCTCAAACAGTTAGTCGTCTCTGGTACCGTCCTGCTTTACCTACCGTAACCGGAAGGGTCAACCTTTAATCCCAt                                                                                                                                  >  1:176307/1‑301 (MQ=255)`  
                                                                                                                                                                           `gCCGTGGGCGCTCGTGGTCTGAACAATCTAGCCTCTAAGCTCATGCTGGCTCTATTCCCTATGCAGACTTGGATGCGACTTACTATATCTGAATATGAAGCAAAGCAGTTACTGAGCGACCCCGATGAACTCGCTAAGGTCGATGAGGGCCTCTCGATGGTAGAGCGTATCATCATGAACTACATTGAGTCTAACAGTTACCGCGTGACTCTCTTTGAGGCTCTCAAACAGTTAGTCGTAGCTGGTAACGTCCTGCTGTACCTACCGGAACCGGAAGGGTCAAACTATAATCCCATGAAGc                                                                                                                             <  1:108448/301‑1 (MQ=255)`  
                                                                                                                                                                                              `tGAACAATCTAGCCTCTAAGCTCATGCTGGCTCTATTCCCTATGCAGACTTGGATGCGACTTACTTTATCTGAATATGAAGCAAAGCAGTTACTGAGCGACCCCGATGAACTCGCTAAGGTCGATGAGGGCCTCTCGATGGTAGAGCGTATCATCATGAACTACATTGAGTCTAACAGTTACCGCGTGACTCTCTTTGAGGCTCTCAAACAGTTAGTCGTAGCTGGTAACGTCCTGCTGTACCTACCGGAACCGGAAGGGTCAAACTATAATCCCATGAAGCTGTACCGATTGTCTTCTTa                                                                                                          <  1:242216/301‑1 (MQ=255)`  
                                                                                                                                                                                              `tGAACAATCTAGCCTCTAAGCTCATGCTGGCTCTATTCCCTATGCAGACTTGGATGCGACTTACTATATCTGAATATGAAGCAAAGCAGTTACTGAGCGACCCCGATGAACTCGCTAAGGTCGATTAGGGCCTCTCGATGGTAGAGCGTATCATCATGAACTACATTGAGTCTAACAGTTACCGCGTGACTCTCTTTGAGGCTCTCAAACAGTTAGTCGTAGCTGGTAACGTCCTGCTGTACCTACCGGAACCGGAAGGGTCAAACTATAATCCCATGAAGCTGTACCGATTGTCTTCTTa                                                                                                          <  1:106308/301‑1 (MQ=255)`  
                                                                                                                                                                                               `gAACAATCTAGCCTCTAAGCTCATGCTGGCTCTATTCCCTATGCAGACTTGGATGCGACTTACTATATCTGAATATGAAGCAAAGCAGTTACTGAGCGACCCCGATGAACTCGCTAAGGTCGATGAGGGCCTCTCGATGGTAGAGCGTATCATCATTAACTACATTGAGTCTAACAGTTATCGCGTGACTCTCTTTGAGGCTCTCAAACAGTTAGTCGTAGCTGGTAACGTCCTGCTGTACCTACCGGAACCGGAAGGGTCAAACTATAATCCCATGAAGCTGTACCGATGGtctgtctct                                                                                                         >  1:311495/1‑295 (MQ=255)`  
                                                                                                                                                                                               `gAACAATCTAGCCTCTAAGCTCATGCTGGCTCTATTCCCTATGCAGACTTGGATGCGACTTACTATATCTGAATATGAAGCAAAGCAGTTACTGAGCGACCCCGATGAACTCGCTAAGGTCGATGAGGGCCTCTCGATGGTAGAGCGTATCATCATGAACTACATTGAGTCTAACAGTTACCGCGTGACTCTCTTTGAGGCTCTCAAACAGTTAGTCGTAGCTGGTAACGTCCTGCTGTACCTACCGGAACCGGAAGGGTCAAACTATAATCCCATGAAGCTGTACCGATTGTCTTCTTAt                                                                                                         >  1:166845/1‑301 (MQ=255)`  
                                                                                                                                                                                                    `aTCTAGCCTCTAAGCTCATGCTGGCTCTATTCCCTATGCAGACTTGGATGCGACTTACTATATCTGAATATGAAGCAAAGCAGTTACTGAGCGACCCCGATGAACTCGCTAAGGTCGATGAGGGCCTCTCGATGGTAGAGCGTATCATCATGAACTACATTGAGTCTAACAGTTACCGCGTGACTCTCTTTGAGGCTCTCAAACAGTTAGTCGTAGCTGGTAACGTCCTGCTGTACCTACCGGAACCGGAAGGGTCAAACTATAATCCCATGAAGCTGTACCGATTGTCGTCTTATGTGGt                                                                                                    >  1:266009/1‑301 (MQ=255)`  
                                                                                                                                                                                                          `ccTCTAAGCTCATGCTGGCTCTATTCCCTATGCAGACTTGGATGCGACTTACTATATCTGAATATGAAGCAAAGCAGTTACTGAGCGACCACGATGAACTCGCTAAGGTCGATGAGGGCCTCTCGATGGTAGAGCGTATCATCATGAACTACATTGAGTCTAATAGTTACCGCGTGACTCTCTTTGAGGCTCTCAAACAGTTAGTCGTAGCTGGTAACGTCCTGCTGTACCTACCGGAACCGGAAGGGTCAAACTATAATCCCATGAAGCTGTACCGATTGTCTTCTTATGTGGTCCAACg                                                                                              <  1:139677/301‑1 (MQ=255)`  
                                                                                                                                                                                                           `ctctAAGCTCATGCTGGCTCTATTCCCTATGCAGACTTGGATGCGACTTACTATATCTGAATATGAAGCAAAGCAGTTACTGAGCGACCCCGATGAACTCGCCAAGGTCGATGAGGGCCTCTCGATGGTAGAGCGTATCATCATGAACTACATTGAGTCTAACAGTTACCGCGTGACTCTCTTTGAGGCTCTCAAACAGTTAGTCGTAGCTGGTAACGTCCTGCTGTACCTACCGGAACCGGAAGGGTCAAACTATAATCCCATGAAGCTGTACCGAttctgtctcttatacacatctccg                                                                                             >  1:152675/1‑279 (MQ=255)`  
                                                                                                                                                                                                           `ctctAAGCTCATGCTGGCTCTATTACCTATGCAGACTTGGATGCGAATTACTATATCTGAATATGAAGCAAAGCAGTTACTGAGCGACCCCGATGAACTCGCTAAGGTCGATGAGGGCCTCTCGATGGTAGAGCGTATCATCATGAACTACATTGAGTCTAACAGTTACCTCGTGACTCTCTTTGAGGCTCTCAAACAGTTAGTCGTATCTGGAAACGTCCTGCTGTACCTACCGGAACCGGAAGGGTCAAACTATAATCCCATGAAGCTGTACCGATTGTCTTCTTATGTGGTCCAACga                                                                                             <  1:313445/301‑1 (MQ=255)`  
                                                                                                                                                                                                             `ctAAGCTCATGCTGGCTCTATTCCCTATGCAGACTTGGATGCGACTTACTATATCTGAATATGAAGCAAAGCAGTTACTGAGCGACCCCGATGAACTCGCTAAGGTCGATGAGGGCCTCTCGATGGTAGAGCGTATCATCATGAACTACATTGAGTCTAACAGTTACCGCGTGACTCTCTTTGAGGCTCTCAAACAGTTAGTCGTAGCTGGTAACGTCCTGCTGTACCTACCGGAACCGGAAGGGTCAAACTATAATCCCATGAAGCTGTACCGATTGTCTTCTTATGTGGTCCAACgaga                                                                                           <  1:255710/301‑1 (MQ=255)`  
                                                                                                                                                                                                               `aaGCTCATGCTGGCTCTATTCCCTATGCAGACTTGGATGCGACTTACTATATCTGAATATGAAGCAAAGCAGTTACTGAGCGACCCCGATGAACTCGCTAAGGTCGATGAGGGCCTCTCGATGGTAGAGCGTATCATCATGAACTACATTGAGTCTAACAGTTACCGCGTGACTCTCTTTGAGGCTCTCAAACAGTTAATCGTAGCTTGTAACGTCCTGCTGTACCTCCCGGAACCGGAAGGGTCAAACTATAATCCCATGTATCTGTACCGAttctttctcttatacacatctccgagcc                                                                                         >  1:56011/1‑275 (MQ=255)`  
                                                                                                                                                                                                                            `ctctATTCCCTATGCAGACTTGGATGCGACTTACTATATCTGAATATGAAGCAAAGCAGTTACTGAGCGACCCCGATGAACTCGCTAAGTTCGATGAGGGCCTCTCGATGGTAGAGCGTATCATCATGAACTACATTGAGTCTAACAGTTACCGCGTGACTCTCTTTGAGGCTCTCAAACAGTTAGTCGTAGCTGGTAACGTCCTGCTGTACCTACCGGAACCGGAAGGGTCAAACTATAATCCCATGAAGCTGTACCGATTGTCTTCTTATGTGGTCCAACGAGACGCATTCGGAAACGt                                                                            >  1:62621/1‑301 (MQ=255)`  
                                                                                                                                                                                                                            `ctctATTCCCTATGCAGACTTGGATGCGACTTACTATATCTGAATATGAAGCAAAGCAGTTACTGAGCGACCCCGATGAACTCGCTAAGGTCGATGAGGGCCTCTCGATGGTAGAGCGTATCATCATGAACTACATTGAGTCTAACAGTTACCGCGTGACTCTCTTTGAGGCTCTCAAACAGTTAGTCGTAGCTGGTAACGTCCTGCTGTACCTACCGGAACCGGAAGGGTCAAACTATAATCCCATGAAGCTGTACCGATTGTCTTCTTATGTGGTCCAACGAGACGCATTCGGCAACGt                                                                            >  1:264213/1‑301 (MQ=255)`  
                                                                                                                                                                                                                            `ctctATTCCCTATGCAGACTTGGATGCGACTTACTATATCTGAATATGAAGCAAAGCAGTTACTGAGCGACCCCGATGAACTCGCTAAGGTCGATGAGGGCCTCTCGATGGTAGAGCGTATCATCATGAACTACATTGAGTCTAACAGTTACCGCGTGACTCTCTTTGAGGCTCTCAAACAGTTAGTCGTAGCTGGTAACGTCCTGCTGTACCTACCGGAACCGGAAGGGTCAAACTATAATCCCATGAAGCTGTACCGATTGTCTTCTTATGTGGTCCAACGAGACACATTCGGAAACGt                                                                            <  1:170403/301‑1 (MQ=255)`  
                                                                                                                                                                                                                            `ctctATTCCCTATGCAGACTTGGATGCGACTTACTATATCTGAATATGAAGCAAAGCAGTTACTGAGCGACCCCGATGAACTCGCTAAGGTCGATGAGGGCCTCTCGATGGTAGAGCGTATCATCATGAACTACATTGAGTCTAACAGTTACAGCGTGACTCACTTTGAGGCTCTCAAACAGTTAGTCGTAGCTGGTAACGTCCGGCTGTACCTACCGGAACAGGAAGGGTCAAACTATAATCTCATGAAGCTGTACCGAGTGGCTTATTCTGTGGTCCAACGAGTCACACTCGGCAAGGt                                                                            >  1:226150/1‑301 (MQ=255)`  
                                                                                                                                                                                                                              `ctATTCCCTATGCAGACTTGGATGCGACTTACTATATCTGAATATGAAGCAAAGCAGTTACTGAGCGACCCCGATGAACTCGCTAAGGTCGATGAGGGCCTCTCGATGGTAGAGCGTATCATCATGAACTACATTGAGTCTAACAGTTACCGCGTGACTCTCTTTGAGGCTCTCAAACAGTTAGTCGTAGCTGGTAACGTCCTGCTGTACCTACCGGAACCGGAAGGGTCAAACTATAATCCCATGAAGCTGTACCGATTGTCTTCTTATGTggctgtctcttatacacatctccgagccc                                                                          >  1:39139/1‑274 (MQ=255)`  
                                                                                                                                                                                                                                     `cTATGCAGACTTGGATGCGACTTACTATATCTGAATATGAAGCAAAGCAGTTACTGAGCGACCCCGATGAACTCGCTAAGGTCGATGAGGGCCTCTCGATGGTAGAGCGTATCATCATGAACTACATTGAGTCTAACAGTTACCGCGTGACTCTCTTTGAGGCTCTCAAACAGTTAGTCGTAGCTGGTAACGTCCTGCTGTACCTACCGGAACCGGAAGGGTCAAACTATAATCCCATGAAGCTGTACCGATTGTCTTCTTATGTGGTCCAACGAGACGCATTCGGCAACGTTCTGCAAAt                                                                   >  1:212691/1‑301 (MQ=255)`  
                                                                                                                                                                                                                                     `cTATGCAGACTTGGATGCGACTTACTATATCTGAATATGAAGCAAAGCAGTTACTGAGCGACCCAGATGAACTCGCTAAGGTCGATGAGGGCCTCTCGATGGTAGAGCGTATCATCATGAACTACATTGAGTCTAACAGTTACCGCGTGACTCTCTTTGAGGCTCTCAAACAGTTAGTCGTAGCTGGTAACGTCCTGCTGTACCTACCGGAACCGGAAGGGTCAAACTATAATCCCATGAAGCTGTACCGATTGTCTTCTTATGTGGTCCAACGAGACGCATTCGGCAACGTTCTGCAAAt                                                                   >  1:58557/1‑301 (MQ=255)`  
                                                                                                                                                                                                                                            `tgtgtataagagacagaCTATATCTGAATATGAAGCAAAGCAGTTACTGAGCGACCCCGATGAACTCGCTAAGGTCGATGAGGGCCTCTCGATGGTAGAGCGTATCATCATGAACTACATTGAGTCTAACAGTTACCGCGTGACTCTCTTTGAGGCTCTCAAACAGTTAGTCGTAGCTGGTAACTTCCTGCTGTACCTACCGGAACCGGAAGGGTCAAACTATAATCCCATGAAGCTGTACCGATTGTCTTCTTATGTGGTCCAACGAGACGCATTCGGCAACGTTCTGCAAATGGTGACt                                                            <  1:219019/285‑1 (MQ=255)`  
                                                                                                                                                                                                                                             `tggttataagagacagcTATATCTGAATATGAAGCAAAGCAGTTACTGAGCGACCCCGATGAACTCGCTAAGGTCGATGAGGGCCTCTCAATGGTAGAGCGTATCATCATGAACTACATTGAGTCTAACAGTTACCGCGTGACTCTCTTTGAGGCTCTCAAACAGTTAGTCGTAGCTGGTAACGTCCTGCTGTACCTACCGGAACCGGAAGGGTCAAACTATAATCCCATGAAGCTGTACCGATTGTCTTCTTATGTGGTCCAATGAGACGCATTCGGCAACGTTCTGCAAATGGTGACTc                                                           <  1:198638/285‑1 (MQ=255)`  
                                                                                                                                                                                                                                                           `tACTATATCTGAATATGAAGCAAAGCAGTTACTGAGCGACCCCGATGAACTCGCTAAGGTCGATGAGGGCCTCTCGATGGTAGAGCGTATCATCATGAACTACATTGAGTCTAACAGTTACCGCGTGACTCTCTTTGAGGCTCTCAAACAGTTAGTCGTAGCTGGTAACGTCCTGCTGTACCTACCGGAACCGGAAGGGTCAAACTATAATCCCATGAAGCTGTACCGATTGTCTTCTTATGTGGTCCAACGAGACGCATTCGGCAACGTTCTGCAAATGGTGACTCGTGACCAGATAGCt                                             >  1:8006/1‑301 (MQ=255)`  
                                                                                                                                                                                                                                                           `tACTATATCTGAATATGAAGCAAAGCAGTTACTGAGCGACCCCGATGAACTCGCTAAGGTCGATGAGGGCCTCTCGATGGTAGAGCGTATCATCATGAACTACATTGAGTCTAACAGTTACCGCGTGACTCTCTTTGAGGCTCTCAAACAGTTAGTCGTAGCTGGTAACGTCCTGCTGTACCTACCGGAACCGGAAGGGTCAAACTATAATCCCATGAAGCTGTACCGATTGTCTTCTTATGTGGTCCAACGAGACGCATTCGGCAACGTTCTGCAAATGGTGACTCGTGACCAGATAGCt                                             >  1:322149/1‑301 (MQ=255)`  
                                                                                                                                                                                                                                                            `aCTATATCTGAATATGAAGCAAAGCAGTTACTGAGCGACCCCGATGAACTCGCTAAGGTCGATGAGGGCCTCTCGATGGTAGAGCGTATCATCATGAACTACATTGAGTCTAACAGTTACCGCGTGACTCTCTTTGAGGCTCTCAAACAGTTAGTCGTAGCTGGTAACGTCCTGCTGTACCTACCGGAACCGGAAGGGTCAAACTATAATCCCATGAAGCTGTACCGATTGTCTTCTTATGTGGTCCAACGAGACGCATTCGGCAACGTTCTGCAAATGGTGACTCGTGACCAGATTGCtt                                            >  1:178750/1‑301 (MQ=255)`  
                                                                                                                                                                                                                                                             `cTATATCTGAATATGAAGCAAAGCAGTTACTGAGCGACCCTGATGAACTCGCTAAGGTCGATGAGGGCCTCTCGATGGTAGAGCGTATCATCATGAACTACATTGAGTCTAACAGTTACCGCGTGACTCTCTTTGAGGCTCTCAAACAGTTAGTCGTAGCTGGTAACGTCCTGCTGTACCTACCGGAACCGGAAGGGTCAAACTATAATCCCATGAAGCTGTACCGATTGTCTTCTTATGTGGTCCAACGAGACGCATTCGGCAACGTTCTGCAAATGGTGACTCGTGACCAGATAGCttt                                           >  1:42608/1‑301 (MQ=255)`  
                                                                                                                                                                                                                                                             `cTATATCTGAATATGAAGCAAAGCAGTTACTGAGCGACCCCGATGAACTCGCTAAGGTCGATGAGGGCCTCTCGATGGTAGAGCGTATCATCATGAACTACATTGAGTCTAACAGTTACCGCGTGACTCTCTTTGAGGCTCTCAAACAGTTAGTCGTAGCTGGTAACGTCCTGCTGTACCTACCGGAACCGGAAGGGTCAAACTATAATCCCATGAAGCTGTACCGATTGTCTTCTTATGTGGTCCAACGAGACGCATTCGGCAACGTTCTGCAAATGGTGACTCGTGACCAGATAGCttt                                           >  1:122218/1‑301 (MQ=255)`  
                                                                                                                                                                                                                                                               `atatCTGAATATGAAGCAAAGCAGTTACTGAGCGACCCCGATGAACTCGCTAAGGTCGATGAGGGCCTCTCGATGGTAGAGCGTATCATCATTAACTACATTGAGTCTAACAGTTACCGCGTGACTCTCTTTGAGGCTCTCAAACAATTAGTCGTAGCTGGTAACGTCCTGCTGTACCTACCGGAACCGGAAGGGACAAACTATAATCCCATGAAGCTGTACCGATTGTCTTCTTATGTGGTACAACGAGACGCATTCGGCAACGTTCGGCAAATGGTGACTCGGGACCAGATAGCTTTTg                                         >  1:89675/1‑301 (MQ=255)`  
                                                                                                                                                                                                                                                               `atatCTGAATATGAAGCAAAGCAGTTACTGAGCGACCCCGATGAACTCGCTAAGGTCGATGAGGGCCTCTCGATGGTAGAGCGTATCATCATGAACTACATTGAGTCTAACAGTTACCGCGTGACTCTCTTTGAGGCTCTCAAACAGTTAGTCGTAGCTGGTAACGTCCTGCTGTACCTACCGGAACCGGAAGGGTCAAACTATAATCCCATGAAGCTGTACCGATTGTCTTCTTATGTGGTCCAACGAGACGCATTCGGCAACGTTCTGCAAATGGTGACTCGTGACCAGATAGCTTTTg                                         >  1:48935/1‑301 (MQ=255)`  
                                                                                                                                                                                                                                                                   `cTGAATATGAAGCAAAGCAGTTACTGAGCGACCCCGATGAACTCGCTAATGTCGATGAGGGCCTCTCGATGGTAGAGCGTATCATCATGAACTACATTGAGTCTAACAGTTACCGCGTGACTCTCTTTGAGGCTCTCAAACAGTTAGTCGTAGCTGGTAACGTCCTGCTGTACCTACCGGAACCGGAAGGGTCAAACTATAATCCCATGAAGCTGTACCGATTGTCTTCTTATGTGGTCCAACGAGACGCATTCGGCAACGTTCTGCAAATGGTGACTCGTGACCAGATAGCTTTTGGTGc                                     >  1:75793/1‑301 (MQ=255)`  
                                                                                                                                                                                                                                                                       `atatGAAGCAAAGCAGTTACTGAGCGACCCCGATGAACTCGCTAAGGTCGATGAGGGCCTCTCGATGGTAGAGCGTATCATCATGAACTACATTGAGTCTAACAGTTACCGCGTGACTCTCTTTGAGGCTCTCAAACAGTTAGTCGTAGCTGGTAACGTCCTGCTGTACCTACCGGAACCGGAAGGGTCAAACTATAATCCCATGAAGCTGTACCGATTGTCTTCTTATGTGGTCCAACGAGACGCATTCGGCAACGTTCTGCAAATGGTGACTCGTGACCAGATAGCtgtctcttataca                                 >  1:258968/1‑289 (MQ=255)`  
                                                                                                                                                                                                                                                                            `aagcaaagcaGTTACTGAGCGACCCCGATGAACTCGCTAAGGTCGATGAGGGCCTCTCGATGGTAGAGCGTATCATCATGAACTACATTGAGTCTAACAGTTACCGCGTGACTCTCTTTGAGGCTCTCAAACAGTTAGTCGTAGCTGGTAACGTCCTGCTGTACCTACCGGAACTGGAAGGGTCAAACTATACTCCCATGAAGCTGTACCGATTGTCTTCTTATGTGGACCAACGAGACGCATTCGGCAACGTTCTGCAAATGGTGACTCGTGAccctgtctcttatacacatctccgagc                            >  1:45307/1‑276 (MQ=255)`  
                                                                                                                                                                                                                                                                               `gatacaggTTACTGAGCGACACCGCTGAACTCGCTAAGGTCGATGAGGGCCTCTCGATGGTAGAGCTTATCATCATGAACTACATTGAATCTAACAGTTACCGCGTGACTCTCTTTGAGGCTCTCAAACAGTTAGTCGTAGCTGGTAACGTCCTGCTGTACCTACCGGAACCGGAAGGGTCAAACTATAATCCCATGAAGCTGTACCGATTGTCTTCTTATGTGGTCCAACGAGACGCATTCGGCAACGTTCTGCAAATGGTGACTAGTGACCAGATAGCTTTTGGTGCTCTCCCTGAGGa                         <  1:308738/294‑1 (MQ=255)`  
                                                                                                                                                                                                                                                                               `caaagcgTTTACTGAGCGACCCCGATGAACTCGCTAAGGTCGATGAGGGCCTCTCGATGGTAGAGCGTATCATCATGAACTACATTGAGTCTAACAGTTACTGCGTGACTCGCTTTGAGGCTCTCAAACAGTTAGTCGTAGCTGGTAACGTCCTGCTGTACCTACCGGAACCGGAAGGGTCAAACTATAATCCCATGAAGCTGTACCGATTGTCTTCTTATGTGGTCCAACGAGACGCATTCGGAAACGTTCTGCAAATGGTGACTCGTGACCAGATAGCTTTTGGTGCTCTCCCTGAGGa                         <  1:29015/301‑1 (MQ=255)`  
                                                                                                                                                                                                                                                                               `caaagcaGTTACTGAGCGACCCCGATGAGCTCGCTAAGGTCGATGAGGGCCTCTCGATGGTAGAGCGTATCATCATGAACTACATTGAGTCTAACAGTTACCGCGTGACTCTCTTTGAGACTCTCAAACAGTTAGTCGTAGCTGGTAACGTCCTGCTGTACCTACCGGAACCGGAAGGGTCAAACTATAATCCAATGAAGCTGTACCGATTGTCTTCTTATGTGGTCCAACGAGACGCATTCGGCAACGTTCTGCAAATGGTGACTCGTGACCAGATAGCTTTTGGTGCTCTCCCTGAGGa                         <  1:252422/301‑1 (MQ=255)`  
                                                                                                                                                                                                                                                                               `aaaagcaGTTACTGAGCGACCCCGATGAACTCGCTAAGGTCGATGAGGGCCTCTCGATGGTAGAGCGTATCATCATGAACTACATTGAGTCTAACAGTTACCGCGTGACTCTCTTTGAGGCTCTCAAACAGTTAGTCGTAGCTGGTAACGTCCTGCTGTACCTACCGGAACCGGAAGGGTCAAACTATAATCCCATGAAGCTGTACCGATTGTCTTCTTATGTGGTCCAACGAGACGCATTCGGCAACGTTCTGCAAATGGTGACTCGTGACCAGATAGCTTTTGGTGCTCTCCCTGAGGa                         <  1:51156/300‑1 (MQ=255)`  
                                                                                                                                                                                                                                                                               `aaaagcaGTTACGGAGCGACCCCGATGAACTCGCTAAGGTCGATGAGGGCCTCTCGATGGTAGAGCGTATCATCATGAACTACATTGAGTCTAACAGTTACCGCGTGACTCTCTTTGAGGCTCTCAAACAGTTAGTCGTAGCTGGTAACGTCCTGCTGTACCTACCGGAACCGGAAGGGTCAAACTATAATCCCATGAAGCTGTACCGATTGTCTTCTTATGTGGTCCAACGAGACGCATTCGGCAACGTTCTGCAAATGGTGACTCGTGACCAGATAGCTTTTGGTGCTCTCCCTGAGGa                         <  1:316825/300‑1 (MQ=255)`  
                                                                                                                                                                                                                                                                                   `gcaGTTACTGAGCGACCCAGATGAACTCGCTAAGGTCGATGAGGGCCTCTCGATGGTAGAGCGTATCATCATGAACTACATTGAGTCTAACAGTTACCGCGTGACTCTCTTTGAGGCTCTCAAACAGTTAGTCGTAGCTGGTAACGTCCTGCTGTACCTACCGGAACCGGAAGGGTCAAACTATAATCCCATGAAGCTGTACCGATTGTCTTATTATGTGGTCCAACGAGACGCATTCGGCAACGTTCTGCAAATGGTGACTCGTGACCAGATTGCTTTTGGTGCTCTCCCTGAggcggtc                     >  1:68378/1‑296 (MQ=255)`  
                                                                                                                                                                                                                                                                                           `tgggCGACCCCGATGAACTCGCTAAGGTCGATGAGGGCCTCTCGATGGTAGAGCGTATCATCATGAACTACATTGAGTCTAACAGTTACCGCGTGACTCTCTTTGAGGCTCTCAAACAGTTAGTCGTAGCTGGTAACGTCCTGCTGTACCTACCGGAACCGGAAGGGTCAAACTATAATCCCATGAAGCTGTACCGATTGTCTTCTTATGTGGTTCAACGAGACGCATTCGGCAACGTTCTGCAAATGGTGACTCGTGACCAGATAGCTTTTGGTGCTCTCACTGAGGACATCCGTAAGGc             <  1:325008/298‑1 (MQ=255)`  
                                                                                                                                                                                                                                                                                            `gAGCGACCCCGATGAACTCGCTAAGGTCGATGAGGGCCTCTCGATGGTAGAGCGTATCATCATGAACTACATTGAGTCTAACAGTTACCGCGTGACTCTCTTTGAGGCTCTCAAACAGTTAGTCGTAGCTGGTAACGTCCTGCTGTACCTACCGTAACCGGAAGGGTCAAACTATAATCCAATGAAGCTGTACCGATTGTCTTCTTAGGTGGTCCAACGAGACGCATTCGGCAACGTTCTGCAAATGGAGACTCGTGACCAGATAGCTTTTGGTGCTCTCACTGAGGCCATCCGTAAGGCt            >  1:156849/1‑301 (MQ=255)`  
                                                                                                                                                                                                                                                                                                 `aCCCCGATGAACTCGCTAAGGTCGATGAGGGCCTCTCGATGGTAGAGCGTATCATCATGAACTACATTGAGTCTAACAGTTACCGCGTGACTCTCTTTGAGGCTCTCAAACAGTTAGTCGTAGCTGGTAACGTCCTGCTGTACCTACCGGAACCGGAAGGGTCAAACTATAATCCCATGAAGCTGTACCGATTGTCTTCTTATGTGGTCCAACGAGACGCATTAGGCAACGTTCTGCAAATGGTGACTCGTGACCAGATAGCTTTTGGTGCTCTCCCTGAGGACATCCGTAAGGCTGTAGa       <  1:132033/301‑1 (MQ=255)`  
                                                                                                                                                                                                                                                                                                 `aCCACGATGAACTCGCTAAGGTCGATGAGGGCCTCTCGATGGTAGAGCGTATCATCATGAACTACATTGAGTCTAACAGTTACCGCGTGACTCTCTTTGAGGCTCTCAAACAGTTAGTCGTAGCTGGTAACGTCCTGCTGTACCTACCGGAACCGGAAGGGTCAAACTATAATCCCATGAAGCTGTACCGATTGTCTTCTTATGTGGTCCAACGAGACGCATTCGGCAACGTTCTGCAAATGGTGACTCGTGACCAGATAGCTTTTGGTGCTCTCCCTGAGGACATCCGTAAGGCTGTAGa       >  1:119957/1‑301 (MQ=255)`  
                                                                                                                                                                                                                                                                                                     `cGATGAACTCGATAAGGTCGATGAGGGCCTCTCGATGGTAGAGCGTATCATCATGAACTACATTGAGTCTAACAGTTACCGCGGGACTCTCTTTGAGGCTCTCAAACAGTTAGTCGTAGCTGGTAACGTCCTGATGTACCTACCGGAACCGGAAGGGTCAAAATATAATCCCATGAAGCTGTACCGATTGTCTTCTTATGTGGTCCAACGAGACGCATTCGGCAACGTTCTGAAAATGGTGACTCGTGACCAGATAGCTTTTGGTGCTCTCCCTGAGGACATCCGTAAGGCTGTAGAAGGt   <  1:114320/301‑1 (MQ=255)`  

                                                                                                                                                                                                                                                                                                          `|`                                                                                                                                                                                                                                                                                                          
`GAGAAACGAACAGGACTTGCGGAGGATGGCGCAAAGTCTGTCTATGAGCGTTTAAAGAACGACCGTGCTCCCTATGAGACACGCGCTCAGAATTGCGCTCAATATACCATCCCATCATTGTTCCCTAAGGACTCCGATAACGCCTCTACAGATTATCAAACTCCGTGGCAAGCCGTGGGCGCTCGTGGTCTGAACAATCTAGCCTCTAAGCTCATGCTGGCTCTATTCCCTATGCAGACTTGGATGCGACTTACTATATCTGAATATGAAGCAAAGCAGTTACTGAGCGACCCCGATGGACTCGCTAAGGTCGATGAGGGCCTCTCGATGGTAGAGCGTATCATCATGAACTACATTGAGTCTAACAGTTACCGCGTGACTCTCTTTGAGGCTCTCAAACAGTTAGTCGTAGCTGGTAACGTCCTGCTGTACCTACCGGAACCGGAAGGGTCAAACTATAATCCCATGAAGCTGTACCGATTGTCTTCTTATGTGGTCCAACGAGACGCATTCGGCAACGTTCTGCAAATGGTGACTCGTGACCAGATAGCTTTTGGTGCTCTCCCTGAGGACATCCGTAAGGCTGTAGAAGGTC  >  NC_001604.1/20246‑20840`

  

Alignment Legend

Aligned base mismatch/match (shaded by quality score): `ATCG/ATCG` `< 3 ≤` `ATCG/ATCG` `< 11 ≤` `ATCG/ATCG` `< 24 ≤` `ATCG/ATCG` `< 35 ≤` `ATCG/ATCG` `< 38 ≤` `ATCG/ATCG`

Unaligned base: `atcg`    Masked matching base: `atcg`    Alignment gap: `‑`    Deleted base: `‑`
